# Automated Affective Computing Based on Bio-Signals Analysis and Deep Learning Approach

**DOI:** 10.3390/s22051789

**Published:** 2022-02-24

**Authors:** Chiara Filippini, Adolfo Di Crosta, Rocco Palumbo, David Perpetuini, Daniela Cardone, Irene Ceccato, Alberto Di Domenico, Arcangelo Merla

**Affiliations:** 1 Department of Neurosciences, Imaging and Clinical Sciences, University G. D’Annunzio of Chieti-Pescara, 9, 66100 Chieti, Italy; chiara.filippini@unich.it (C.F.); david.perpetuini@unich.it (D.P.); d.cardone@unich.it (D.C.); irene.ceccato@unich.it (I.C.); 2 Department of Psychological, Health and Territorial Sciences, University G. D’Annunzio of Chieti-Pescara, 9, 66100 Chieti, Italy; adolfo.dicrosta@unich.it (A.D.C.); rocco.palumbo@unich.it (R.P.); alberto.didomenico@unich.it (A.D.D.)

**Keywords:** affective computing, emotion recognition, infrared imaging, thermal imaging

## Abstract

Extensive possibilities of applications have rendered emotion recognition ineluctable and challenging in the fields of computer science as well as in human-machine interaction and affective computing. Fields that, in turn, are increasingly requiring real-time applications or interactions in everyday life scenarios. However, while extremely desirable, an accurate and automated emotion classification approach remains a challenging issue. To this end, this study presents an automated emotion recognition model based on easily accessible physiological signals and deep learning (DL) approaches. As a DL algorithm, a Feedforward Neural Network was employed in this study. The network outcome was further compared with canonical machine learning algorithms such as random forest (RF). The developed DL model relied on the combined use of wearables and contactless technologies, such as thermal infrared imaging. Such a model is able to classify the emotional state into four classes, derived from the linear combination of valence and arousal (referring to the circumplex model of affect’s four-quadrant structure) with an overall accuracy of 70% outperforming the 66% accuracy reached by the RF model. Considering the ecological and agile nature of the technique used the proposed model could lead to innovative applications in the affective computing field.

## 1. Introduction

Over the past few years, the study of emotion recognition has attracted growing interest, and presents an increasing trend in computer science. Emotion prediction and recognition play a vital role in various domains such as digital multimedia entrainment, self-driving, healthcare, and human–computer interface [1]. Indeed, it has been shown that communication between humans and computers benefits from sensor-based emotion recognition since humans experience discomfort when emotions are absent [2]. Moreover, Reeves et al. stated that people treat computers the same way they treat people [3]. As a result, computers should show empathy to their users. 

Emotions are further essential for motivation and learning [4]. During computer-assisted learning, affective interaction could be beneficial and improve one’s mental state. In this regard, on the artificial system’s side, emotion recognition improves communication efficiency between the machine and its user while also assisting in achieving the intended goal. Moreover, emotion recognition would benefit more than human–computer interactions. For instance, it would also help psychologists identify the emotions of individuals who have difficulties expressing their feelings [5,6,7,8,9]. Furthermore, the healthcare field is becoming increasingly reliant on technological devices and applications [10]. Patients’ motivation may be improved by interacting with a virtual avatar that can read and tune to the users’ emotions [11]. In rehabilitative applications, this may result in a faster and more successful recovery and improved quality of life [12]. However, while highly desirable, an accurate and automated emotions classification approach remains a challenging problem in the affective computing field.

To avoid mistakes in emotion identification and to design a reliable setup, a deeper understanding of emotion modeling, processing, and expression is necessary. Research in psychology and neuroscience has been markedly interested in exploring the underlying structure of emotions and affective experience [13]. Based on the categorical approach to the study of emotions, psychologists considered basic emotions as discrete entities characterized by a fixed expression of nerves and bodily functions, corresponding to singular emotional expression and feeling [14]. In this context, the Basic Emotion Theory (BET) described affect as a set of singular phylogenetically and ontogenetically primary states (such as happiness, sadness, fear, disgust, surprise, etc.), which are considered independent of each other and emerging from distinct neural systems [14,15,16]. However, recent findings in neuroscience and behavioral genetics have highlighted consistent difficulties in identifying and describing emotions within the categorical approach [17]. Additionally, evidence suggests that humans do not perceive or recognize emotions as distinct entities but rather as ambiguous and overlapping experiences [18]. These considerations have prompted authors to adopt a dimensional approach in the study of emotions, considering the distinct affective states arising from common and interconnected neurophysiological systems [19]. Specifically, the dimensional approach allows for a classification of emotions based on underlying fundamental dimensions beyond the particular types of emotional response [20]. One of the most studied dimensional models is the circumplex model of affect (CMA) [21]. This model proposes that all affective states can be interpreted as a linear combination of two fundamental dimensions: valence and arousal. The valence dimension, varying on a pleasure–displeasure continuum, describes the hedonic tone or the degree to which an emotion is pleasant or unpleasant. The arousal dimension refers to the level of psychological activation induced by an emotion [22,23]. According to the CMA, from the linear combination of the different levels of both valence (e.g., positive/negative) and arousal (e.g., high/low), it is possible to organize and interpret all the discrete emotions [17,21]. For instance, “happiness” is an emotional state resulting from a significant activation in the neural systems associated with positive valence, combined with a moderate activation in the neural systems associated with arousal [24]. 

**Figure 1:**
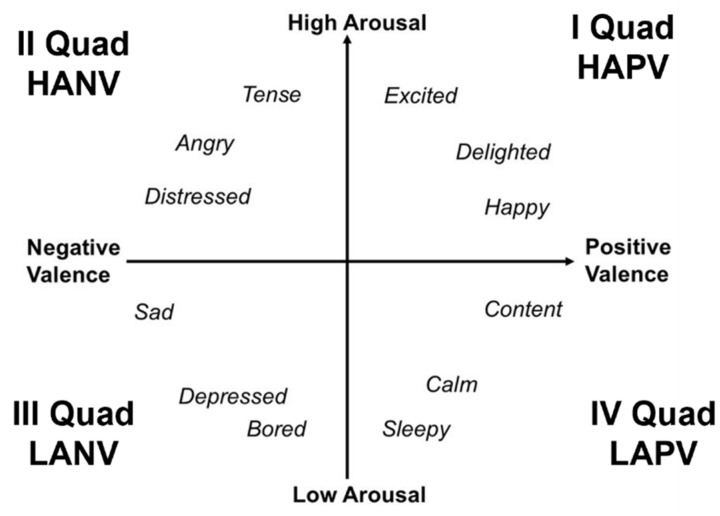
Graphical representation of the circumplex model of affect (CMA) with the horizontal axis representing the valence dimension and the vertical axis representing the arousal dimension (adapted from [30]). The first CMA’ quadrant (I Quad) is representative of High Arousal and Positive Valence (HAPV), the second quadrant (II Quad) correspond to High Arousal and Negative Valence (HANV), the third quadrant (III Quad) to Low Arousal and Negative Valence (LANV), and the fourth quadrant (IV Quad) to Low Arousal and Positive valence (LANV).

In this study, the human emotional states were classified into four classes based on the four quadrants of the CMA [17]. These four quadrants are determined by the respective levels on both valence and arousal dimensions, as follows: (1) high arousal (HA)—positive valence (PV); (2) high arousal (HA)—negative valence (NV); (3) low arousal (LA)—negative valence (NV); (4) low arousal (LA)—positive valence (PV). A graphical representation of the four-quadrant structure of the CMA developed by Russell [21] is depicted in Figure 1. Importantly, recent studies considered Russell’s circumplex model in the development of an extensive range of procedures and experimental materials useful to the study of emotions [21]. In this context, several authors developed validated databases, which indexed a large set of different emotional stimuli based on the valence and the arousal dimensions. These databases mainly differ from each other in the type of emotional stimuli used, including pictures (e.g., IAPS; [25]), words (e.g., ANEW; [26]), sounds (e.g., IADS; [27]), or videos (e.g., CAAV; [28]). In this study, we employed video stimuli extrapolated from the Chieti Affective Action Video (CAAV) database [28,29] to analyze individuals’ behavioral and physiological responses associated with the different pattern levels of valence and arousal expressed from the video stimuli.

Furthermore, up to now, the emotion recognition methods are classified into trait-based (i.e., facial expression, gestures, and speech) and physiological-state-based approaches [31]. Although the former have been majorly explored and used for emotion classification purposes [32], they may be prone to subconscious control, resulting in inaccurate performance. On the contrary, approaches based on physiological signals (which cannot be voluntarily altered) can reflect people’s emotions more accurately than non-physiological ones [33] and are growing increasingly prominent. This is also due to the fact that internet of things (IoT) technology has recently rendered physiological data widely available. Indeed, owing to the rapid developments of flexible electronics, IoT, machine learning (ML), and cloud computing, wearable devices have become a powerful tool for people’s daily health monitoring due to their convenience and unobtrusive everyday use [34]. People are interested in purchasing smart devices to monitor their health, such as heart rate, blood pressure, and mobility. Moreover, this technology can be used to detect emotions, thus providing potential improvement for emotion monitoring and regulation. Indeed, emotions influence the activity of the Autonomous Nervous System (ANS), which in turn regulates various body parameters such as heart rate variation, respiration, and temperature patterns.

Existing approaches of emotion classification based on physiological signal analysis have largely depended on the study of only one, a few, or isolated physiological signals and have often employed expensive and uncomfortable sensors to be used in real-life environments. However, as stated in Shu et al., using multiple signals in an emotion recognition system greatly improves the overall accuracy and presents its significance in both research and real applications [35]. 

Among the prominent physiological signals triggered during the emotional response, cardiac-related parameters such as heart rate variability (HRV) [36] as well as blood volume pulse (BVP) have received the greatest attention for emotion detection purposes [37]. In addition, respiration (RSP) patterns provide important information about the individual’s emotional condition [38]. Therefore, in this study, HRV, BVP, and RSP were assessed to differentiate the emotional states of the individual. Of particular interest, these signals can be easily collected with smart devices or IoT technologies, allowing the development of an emotion recognition model for use in real-world applications. A point of innovation introduced in this study is the combined use of such signals with a smart and non-contact system able to monitor physiological parameters such as skin temperature modulation, i.e., thermal infrared (IR) imaging. The use of IR imaging has increased to analyze a range of problems in different fields of human knowledge. In recent years, different emotions have been studied through IR imaging as when an emotion occurs, a change in facial temperature appears [39,40]. This is mostly due to the blood flow that the body emits through blood vessels in the subcutaneous facial area; such a change can be qualified and quantified through IR imaging [41,42]. Furthermore, IR imaging can be easily interfaced within an IoT context due to its contactless and smart nature.

Finally, the process of constructing the emotion recognition model includes data collection, emotion-related feature extraction, and classifier model building. To develop the classification model, machine learning (ML) approaches have been applied. ML discipline deals with the development and analysis of algorithms capable of learning from data [43]. Such algorithms operate by fostering a model based on inputs and using these inputs to make predictions or decisions [44]. In the context of emotion detection, ML algorithms are used to learn methods for detecting emotions. There are two types of learning approaches: supervised and unsupervised learning. The supervised learning algorithm analyses the training data and derives a function that can be employed for mapping new examples. It is called supervised learning since the classification process is guided by the labeled training set. In this study, a random forest (RF) supervised ML classifier was used. Indeed, based on recent empirical studies and performance comparisons of different ML-based classifiers, RF outperforms other types of ML classifiers in terms of generalization ability [45,46]. 

Within ML, in recent years, deep learning (DL) approaches have achieved tremendous success in various domains. Different DL architectures have emerged as effective models across numerous tasks. Feedforward neural networks (FFNNs) are the most widely used and arguably most simple type of DL devised. An FFNN represents a biologically inspired classification algorithm consisting of a number of neuron-like processing units. Units are organized in layers, and every unit is connected with all units in the previous layer. Therefore, the information travels in only one direction forward: from the input nodes, data traverse through the hidden nodes to the output nodes. 

In the present study, in order to develop an accurate and automated emotion classification system, both conventional ML approaches, such as RF algorithm, and recent DL approaches, such as FFNN, were developed and compared. The implemented classifiers rely only on involuntary physiological signals easily collected by smart devices. The novelty introduced in this study is the combined use of such signals with a smart and non-contact technology i.e., thermal IR imaging. Indeed, the automated emotion recognition model was designed with the aim of being implemented in IoT and smart technology contexts, as well as in real-life affective computing tasks. Furthermore, the salient features of each physiological signal were identified and statistically assessed. The presented approach to emotion classification is highly suitable to the field of affective computing and human–machine interaction and may be used to drive improved evaluation, monitoring, and intervention in emotional processing and regulation.

## 2. Materials and Methods

### 2.1. Participants

The sample was composed of 50 Italian adults from 18 to 35 years old (mean = 22.80, standard deviation = 3.11, women = 65.0%) without self-reported psychiatric illnesses or severe mood disorders. All participants were right-handed as assessed with the Manual Preference Questionnaire [47]. All participants were university students at G. d’Annunzio University of Chieti-Pescara. The mean education level, measured in years, was 16.62 (standard deviation = 2.58). The Ethical Committee of the Department of Psychological, Health and Territorial Sciences at G. d’Annunzio University of Chieti-Pescara (protocol number: 20018) approved the study. All participants provided their written consent to participate. They received no monetary or other forms of compensation for their participation.

### 2.2. Emotional Stimuli Dataset

The present study aims to analyze behavioral and physiological responses associated with the presentation of a series of stimuli, characterized by different pattern levels on both valence and arousal dimensions. To this aim, we selected emotional video stimuli extrapolated from the CAAV database [28,29]. This database consists of a series of emotional video stimuli developed specifically for experimental research. All video stimuli videos lasted 15 *s* and included everyday life actions. The CAAV is based on the assumption that using videos to elicit emotional reactions could offer different advantages than employing other more simple static visual stimuli such as pictures or words [48,49]. Moreover, CAAV was founded based on the CMA and, therefore, for each stimulus, the authors provided two emotional rating scores, respectively, based on valence and arousal dimensions. In our study, 12 videos extrapolated from the CAAV were selected. These videos were chosen based on their rating scores on the valence and arousal dimensions to obtain two videos for each of the four quadrants of the CMA developed by Russell [21]. In detail, the videos associated with NV (M = 3.17, SD = 0.12) and LA (M = 4.76, SD = 0.08) were named “Throwing a ring on the ground” and “Seeing a pair of broken glasses”. The videos associated with NV (M = 2.18, SD = 0.07) and HA (M = 6.38, SD = 0.43) were named “Losing Hair” and “Finding a fly on the plate”. The videos associated with PV (M = 6.57, SD = 0.31) and LA (M = 3.8, SD = 0.23) were named “Solving a puzzle” and “Making soap bubbles”. The videos associated with PV (M = 7.20, SD = 0.27) and HA (M = 5.76, SD = 0.22) were named “Eating pizza” and “Finding cash”. Furthermore, we selected four other videos (“Drawing a triangle”, “Buttoning a shirt”, “Wearing a jacket”, “Closing an umbrella”) defined as neutral based on the valence parameter (M = 5.15, SD = 0.18) and with low arousal (M = 2.28, SD = 0.35) as control stimuli. Finally, we used two practice videos of the CAAV for the training phase. 

### 2.3. Procedure

Experimental sessions were conducted individually in a laboratory room, maintaining a fixed temperature of 23 ± 1 °C; relative humidity at 50–55% with no direct ventilation on the participant, no direct sunlight, and similar lighting conditions. Participants were asked to sit in front of a computer screen and maintain a distance from the screen of about 60 cm for the entire duration of the experimental task. Before starting the task, two experimenters set up the assembly and the calibration of the systems for recording physiological and thermal signals. Following a 10-min acclimatization phase in the room, participants were administered the experimental task via the E-prime 3.0 software. This software was used to randomize the presentation of the video stimuli, to collect participants’ responses during the rating phase, as well as to control the timing, and to trigger the signals during the different experimental phases. Specifically, the experimental procedure started with a period of 15 s, recorded as a baseline, in which a fixation cross appeared on the screen. Afterward, participants were asked to carefully watch a CAAV video for its entire duration (15 s). After 15 s resting state in which another fixation cross appeared on the screen, they were asked to rate the video on valence and arousal dimensions. As in the validation of the CAAV database, we used two 9-point SAM scales for this rating phase [50]. Regarding the SAM scales, each number corresponds to a figure representing the different emotional reactions associated with the levels of either valence or arousal. A score of 1 corresponds to the lowest possible rating (either NV or LA), and a score of 9 corresponds to the highest possible rating (either PV or HA). Participants select their responses by pressing the corresponding numeric key on the computer keyboard. A standard 5 s rating period was used for each dimension. The order of the presentation of these two scales was randomized across the experimental participants. At the end of the rating phase, the entire procedure started again, and a new video was presented on the screen. All participants were presented with 12 CAAV videos during the experimental session. The entire procedure lasted approximately 15 min.

### 2.4. Physiological Signals Acquisition and Processing

Emotional states are associated with detectable ANS physiological responses. These responses can be collected via body-worn physiological sensors such as electrocardiography (ECG), photoplethysmography (PPG), and a respiratory belt, as well as contactless sensors such as thermal IR cameras. To gather HRV, BVP, and RSP data, the Encephalan Mini AP-10 data acquisition wireless system was employed. The system is equipped with ECG, PPG sensors, and a respiratory belt, and allows multichannel wireless acquisition. The system sampling frequency is 250 Hz. 

The facial skin temperature was recorded by means of a digital thermal IR camera FLIR A655 (FLIR, Wilsonville, OR, USA) (640 × 480 bolometer FPA, sensitivity/noise equivalent temperature difference: <30 mK @30 °C, field of view: 24° × 18°). The sampling frequency was set to 6Hz. The camera was blackbody-calibrated in order to remove eventual drift/shift of the sensor’s response and optical artifacts. In accordance with the literature, cutaneous emissivity was considered as ε = 0.98 [51]. To reduce skin temperature variability due to environmental conditions, the experimental room temperature and relative humidity were kept fixed at 23 ± 1 °C; and relative humidity at 50–55%. The acquisitions were performed in accordance with the standard guidelines for thermal measurements [52]. Moreover, the participants were asked to adhere to a preparation protocol, which included refraining from vigorous exercise, caffeine, and alcohol for 4 h prior to the experimental session as well as refraining from using moisturizing cream and make-up [53]. The thermal device was directed toward the participant’s face at a distance of 60 cm. Participants were instructed to look straight ahead. However, slight participants’ head rotation (in range 75–105 °C between the thermal camera focal plane and the participants’ head) can be considered acceptable [54]. Other than thermal imaging, visible imaging was also recorded through a Logitech C920 HD Pro webcam with 1920 × 1080 video resolution.

#### 2.4.1. Cardiac Features: Heart Rate Variability and Blood Volume Pulse 

**Figure 2:**
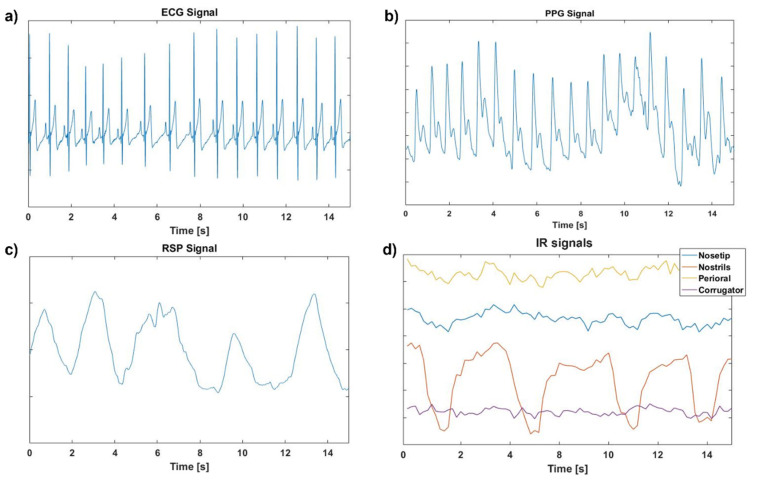
Pre-processed physiological signals trends during the task of a randomly chosen participant. In detail, (**a**) Electrocardiogram, (**b**) Photoplethysmogram, (**c**) Respiration, and (**d**) Thermal infrared signals trends.

Heart rate variability (HRV) refers to the fluctuations between consecutive heartbeat cycles. It is usually represented by the variation in the heart rate’s beat-to-beat temporal changes (RR intervals). HRV has been regarded as an objective measure of emotional response, among which the polyvagal theory and the model of neurovisceral integration represent the main supporting theories [55]. A wide range of publications have demonstrated that the traditional time-domain and frequency-domain indices of HRV are able to characterize autonomic activity among emotions. The time interval of the QRS wave or the instantaneous heart rate at a specific time period is evaluated in time-domain analysis. Conversely, frequency domain analysis is employed to analyze the power distribution of the frequency function and to quantify the autonomic nervous system balance [56]. Therefore, the features extracted for time-domain analysis were the mean RR interval, the standard deviation of normal-to-normal interval (SDNN), and root-mean-square of successive RR interval differences (RMSSD). The formulas of such indices are reported in Table 1. In addition, features such as low frequency (LF), high frequency (HF), and LF/HF ratio were assessed through frequency-domain analysis. In detail, the LF indicates the activity of the sympathetic nervous system, the HF indicates the activity of the parasympathetic nervous system, and the LF/HF ratio [42] is used to determine the ratio of sympathetic and parasympathetic activity (Table 2). Prior to feature extraction, the ECG signal was pre-processed. Specifically, the ECG was carefully inspected to identify potential artifacts, ectopic beats, and arrhythmic events. Since HRV analysis relies on the sinus rhythm, these artifacts and non-sinus events would introduce errors if left untreated. Therefore, non-sinus events were deleted or replaced by linear and cubic spline interpolation. Moreover, a 3rd order Butterworth filter was used to remove the effects of noise and baseline wander from the ECG signals with cut-off frequencies of 0.02 Hz and 17 Hz, in accordance with Chen et al. [57] (Figure 2a). Finally, the ECG peaks were identified considering the local maxima on the filtered signal. In order to improve the accuracy of the procedure, some constraints were defined: the minimum value of the peak was set at 0.5 mV [57] and the temporal interpeak distance was set at 600 ms, which is compatible with the human heart rate at rest. A visual inspection showed a 100% accuracy of the method. Identification of the peaks allowed evaluation of the different metrics associated with HRV.

A novel technique, preferable for mobile use, is measuring the BVP by PPG, which is extensively used in smartwatches. PPG enables infrared light to pass through tissue and measure light absorption by blood flowing through blood vessels beneath the skin. It can be employed to detect the heartbeat’s blood flow rate, also known as BVP. The BVP is regulated by the ANS and recognized as a valuable information source for emotion recognition [58]. The features extracted from the signal and used for emotion detections were the standard deviation (STD), the signal average value (Mean), the signal variation (Δ) (i.e., the difference between the average value of the signal during task and rest), and the sample entropy (SampEn). Sample Entropy is a useful tool for investigating the dynamics of heart rate and other time series. It evaluates the non-linear predictability of a signal, its regularity, and complexity (Table 2). 

Prior to feature extraction, the PPG signal was pre-processed in accordance with the procedure described by Perpetuini et al. [59]. In detail, the signal was corrected for motion artifacts and filtered with a zero-lag, 3rd order, band-pass Butterworth digital filter (cut-off frequencies: 0.2 and 10 Hz) (Figure 2b). The PPG signal was collected employing the Encephalan PPG sensor placed on the index finger. 

#### 2.4.2. Respiration Features

RSP is primarily controlled for metabolic and homeostatic purposes. However, autonomic breathing is not only regulated by metabolic demands but also consistently responds to changes in emotions. The brainstem and higher centers, including the limbic system and cortical regions, interact to produce the final respiratory output [60]. Furthermore, RSP bears a dominant influence on the HF component of the HRV since an individual’s heart rate increases during inspiration and decreases during expiration, a condition known as respiratory sinus arrhythmia. Moreover, RSP pattern, velocity, and depth contain rich information about emotional states [38]. It is, indeed, interesting that respiration, which is essential in maintaining physiological homeostasis, and emotions coexist [60]. 

To characterize the emotional response from the RSP signal, features such as STD, Mean, Δ, and SampEn were extracted and used as input for the ML classifier. Prior to feature extraction, the RSP signal was pre-processed by filtering out noise and artifacts (Figure 2c). Specifically, the RSP signal was filtered by a 3rd order, band-pass Butterworth digital filter from 0.04 Hz to 0.8 Hz in accordance with Valderas et al. [61]. The Encephalan respiratory belt sensor was used to gather the RSP signal. Table 2 contains an explanation of the features employed for each physiological signal.

#### 2.4.3. Thermal Infrared Imaging Features

IR imaging allows contact-less and non-invasive recording of the cutaneous temperature and its topographic distribution through the measurement of spontaneous body thermal irradiation. By recording the temperature dynamics in specific facial regions of interest (ROIs), it is possible to identify peculiar features correlated to emotional state and measures associated with standard physiological signals of the sympathetic and parasympathetic activity [40]. Indeed, as mentioned, the observations of affective nature derive primarily from changes in breathing or heart rate as well as from subcutaneous blood flow, which are all phenomena controlled by the ANS. Vasomotor processes can be identified and monitored over time as they produce a thermal variation of the skin. Vasoconstriction occurs mainly in threat response, and it implies a decrease in temperature due to the restriction of the blood flow to the surface of the skin. By contrast, once the thread has been faced, vasodilatation is observed along with a gradual temperature rise due to parasympathetic restoration. 

As a body area of interest, for thermal inference of an affective nature, the human face is considered of particular importance since it can be easily recorded and is naturally exposed to social and emotional interaction. In particular, ROI such as nose tip, nostrils, glabella (corrugator muscle region), and perioral areas, resulted as the most reliable regions for the detection of the autonomic activity [62,63]. Therefore, these four ROIs were selected and assessed in this study. 

**Figure 3:**
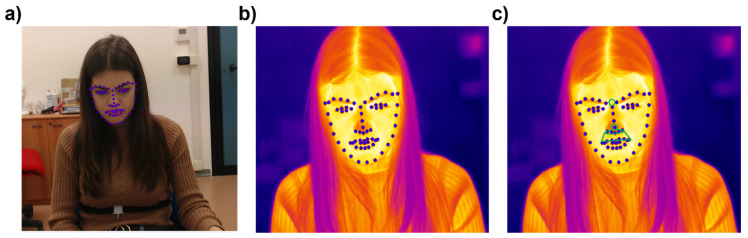
Thermal tracking pipeline. (**a**) Facial landmarks’ automatic recognition in the visible domain. (**b**) Facial landmarks’ identification on the corresponding IR images. (**c**) ROI identification with respect to the facial landmark. The green circles correspond to the four facial regions tracked, which are: the nose tip, nostrils, perioral, and corrugator area.

The thermal camera was associated with a Logitech C920 webcam located above it for the acquisition of the visible videos. Visible and IR videos of the participants’ faces were simultaneously recorded during the experiment. For all participants, the time series of facial IR images were visually inspected to ensure adequate quality of the recordings. The variations in facial ROIs’ cutaneous temperature were then assessed using customized programs. Since the participants were free to move their heads without any restriction, a soft-tissue tracking algorithm was developed to track the selected ROIs throughout all images of the time-series in order to accurately compute the ROIs temperature from each facial thermogram. The tracking algorithm relied on facial landmarks detection over time and was developed in Python in accordance with the procedure described by Cardone et al. [64]. Given the availability of computer vision algorithms for visible video, in the present study, visible images were employed as a reference for tracking facial landmark features. The tracking algorithm was indeed based on the following processes steps:

- Co-registration between visible and thermal optics. The co-registration was performed by applying a geometric transformation of the visible coordinates (calculated based on different fields of view), resolution, and position.
- Facial landmarks’ automatic recognition in the visible domain using the OpenFace library. OpenFace is an open-source tool, able to detect facial landmarks, estimate head pose, recognize facial action units, and estimate eye-gaze on standard RGB images [65] (Figure 3a).
- Facial landmarks’ identification on the corresponding IR images by applying the geometrical transformation obtained from the optical co-registration process (Figure 3b).
- ROI identification with respect to the facial landmark in the IR images (Figure 3c)

For each participant, the tracking algorithm permitted collection of the ROIs’ average temperatures pattern across all episodes of the experimental paradigm. The ROIs’ IR signals were preprocessed prior to feature extraction. Specifically, the IR signals were corrected for residual motion artifacts. Motion errors were identified and replaced by performing a linear interpolation of the value neighboring the artifact. The corrected ROIs IR signals were then filtered by a 3rd order, low-pass Butterworth digital filter at 1Hz (Figure 2d). Five representative features were computed from the pre-processed IR signals of each ROI, as described in Table 2. These were: STD, Mean, Δ, SampEn, Der1.

### 2.5. Classification Algorithms 

#### 2.5.1. Machine Learning—Random Forest

Firstly, an ML approach was used to classify the participant’s emotional state relying on features extracted from their physiological signals. Specifically, the participant’s emotional condition was classified into four classes related to the four CMA’s quadrants (i.e., HAPV, HANV, LAPV, LANV). These classes were identified based on the SAM outcome. The median value of the overall arousal and valence distributions, respectively, were utilized to split the arousal into high or low and the valence into positive or negative. 

As an ML approach, the RF algorithm was employed and trained through a supervised learning procedure. RF is an ensemble method with a composite structure. The classification is performed using a forest of decision trees, each constructed using a random subset of the features [66]. In detail, the RF classifier predicts the class label of input data by majority voting on the predictions rendered by the set of tree classifiers. The RF was trained on z-scored data. Owing to the multivariate (34 input features (Table 2)) RF approach, in-sample performance of the procedure did not efficiently estimate the out-of-sample performance. A cross-validation procedure was, thus, implemented to assess the procedure’s generalization capabilities. Specifically, a leave-one-subject-out cross-validation was performed [53]. This cross-validation approach consisted of leaving one subject (precisely all samples from the same participant) out of the classification and in classifying the predicted output value on the given subject using the other participants as the training set of the RF model. This procedure was iterated for all participants, and further statistical analyses were performed on the out-of-training-sample classification of the participants’ emotional states. Finally, in order to evaluate the advantage of the multivariate procedure, the performance of the cross-validated multivariate approach, based on both cardiac (i.e., HR, BVP), respiratory (RSP), and thermal (IR) features, was compared to that of the classifier based only on cardiac features, as well as to that of the classifier based on both cardiac and respiratory characteristics together. The performances of all approaches used were compared employing the McNemar–Bowker test. 

#### 2.5.2. Deep Learning—Feed Forward Neural Network 

**Figure 4:**
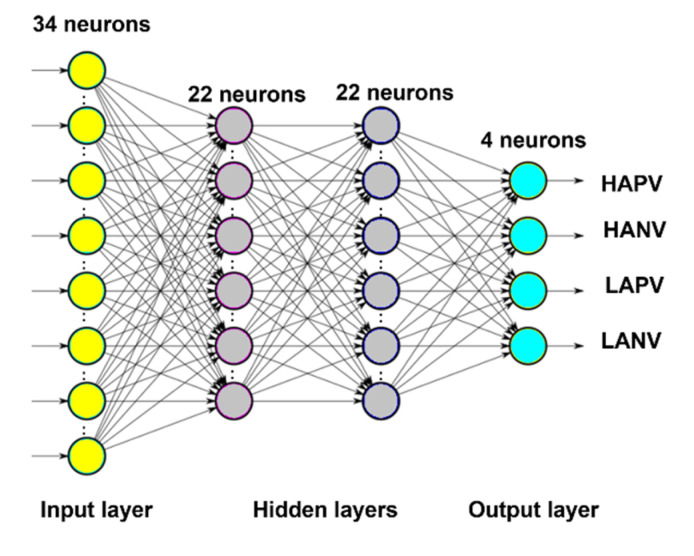
First Feed Forward Neural Network architecture.

While FFNNs are fairly straightforward, their simplified architecture was leveraged to advantage in several ML applications. The FFNNs accept multiple inputs and multiply each input by a weight before summing the results. The weights bear the function to enhance or inhibit the input signal, thus simulating the function of synapses in biological neurons. A bias value is then added to the result before it is passed to an activation function. The FFNN employed in this study was trained with a supervised learning procedure. Such a procedure involves the use of a set of input–output pairs, called the training set, in conjunction with a training algorithm that iteratively adjusts model parameters (i.e., weights and biases) depending on the training set, allowing the model to accurately map inputs to outputs. The implemented FFNN comprised an input layer, two hidden layers, and an output layer. The input layer contained as many neurons as the dimension of the input data, i.e., 34 neurons. The first and second hidden layers were composed of 22 and 22 neurons, respectively. The output layer consisted of 4 neurons related to the 4 classification classes (i.e., HAPV, HANV, LAPV, LANV). The FFNN architecture is shown in Figure 4. The activation function of the input and hidden layers was the Rectified Linear Unit (ReLu) function. Such an activation function was chosen since it was demonstrated to suffer less from the vanishing gradient problem [67]. Furthermore, a SoftMax function was used in the output layer to output a probability value from 0 to 1 for each of the four classification classes. Finally, a loss function must be included to objectively quantify how much the network’s predicted output differs from the expected output (the associated label). In this study, a categorical cross-entropy function was used as a loss function. The FFNN was trained on z-scored data.

An optimization approach was then implemented to improve the developed architecture. Specifically, the optimization procedure was mainly dedicated to decreasing overfitting by employing a regularization approach [68]. The dropout regularization method was used in this study; during training, neurons were randomly deleted from the neural network. This prevented FFNN from over-co-adapting to the testing set. Moreover, to address the model generalization performance, a leave-one-subject-out cross-validation procedure was performed. Finally, rather than employing a fixed learning rate hyperparameter, which might cause the model to converge to a suboptimal solution too rapidly, a tunable learning rate was performed throughout the training phase. Specifically, a function was employed to reduce the learning rate by a factor of 0.1 once learning ceased improving after a minimum of 5 epochs. 

The optimization procedure was iterated for 700 epochs, with a batch size of 9 samples. The model was assessed using the accuracy metric. The accuracy describes the percentage of correct predictions out of the total number of test samples. In detail, after an argmax evaluation of the FFNN output vector, the accuracy metrics were calculated by counting the number of correct FFNN predictors and averaging them among plateau iterations. The described FFNN model was implemented in Python utilizing the Keras API with the TensorFlow backend. The scikit learn library was used to evaluate the model.

The first layer’s weights were retrieved from the developed FFNN model, and their absolute values were computed to identify the salient features used by the FFNN to reach its final classification outcome. The features associated with the highest weights for each physiological signal were further analyzed through a canonical statistical analysis. Such analysis was performed to investigate the presence of a statistical difference between their trend across the four emotional conditions. Specifically, a one-way analysis of variance with repeated measures (ANOVA RM) was computed on each salient feature, with the four classes as a within-subject factor. Prior to the ANOVA RM implementation, a test for data normality and sphericity was carried out through the Shapiro–Wilk test and Mauchly’s test, respectively. All data met the assumptions. Following the ANOVA, RM Student’s t-test was used to compare pairs of means. 

## 3. Results

### 3.1. Random Forest Classification Results 

**Figure 5:**
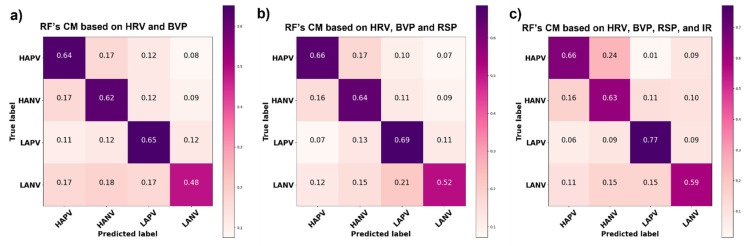
Normalized confusion matrix of (**a**) Random Forest emotion classification based on cardiac features, (**b**) Random Forest emotion classification based on cardiac and respiratory features, and (**c**) Random Forest classification outcome relied on cardiac, respiratory, and thermal features.

The classification performance was assessed using the confusion matrix (CM) on each of the four output classes (i.e., HAPV, HANV, LAPV, LANV). A CM represents a performance metric commonly employed in ML classification tasks. Such a matrix compares the expected values to those predicted by the model under consideration. This offered a comprehensive picture of how well the classification model was performing and what types of mistakes it was producing. The CM related to the RF emotion classification based on the cardiac features (i.e., HRV and BVP) is reported in Figure 5a, the overall accuracy reached in this classification measured 59.5%. Figure 5b reports the CM related to the RF classification based on cardiac, and respiratory features, the overall accuracy achieved was 63%. Finally, in Figure 5c, the CM related to the RF classification based on cardiac, respiratory, and thermal features is reported, the overall accuracy reached in the classification measured 66%. 

To test the statistical significance of the differences in the performances of the classifiers, a McNemar–Bowker test was performed on the classifiers’ prediction outcome. Indeed, such a test allowed the comparison between more than 2 classes–classifiers. Although the multivariate approach achieved higher accuracy, no statistical significance was identified between the performance of the classifier based only on the cardiac features and the classifiers based on cardiac and respiratory features (B = 0.8 p = n.s) as well as between the performance of the classifier based only on the cardiac features and the classifiers based on cardiac, respiratory and thermal features (B = 2.7, p = n.s.)

### 3.2. FeedForward-Neural Network Classification Results 

**Figure 6:**
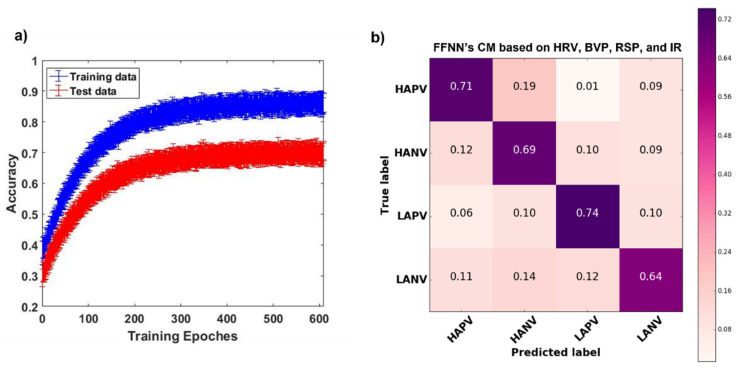
(**a**) FeedForward Neural Network average (and related standard error) cross-validated accuracy as a function of the training epoch for training and testing set, respectively. (**b**) Normalized confusion matrix related to the FeedForward Neural Network emotional classifiers based on cardiac, respiratory, and thermal features.

The accuracy trend of the FFNN model as a function of training epochs for training and test set is reported in Figure 6a. The model did not show any overfitting effect. The average accuracy achieved a plateau value of 70% ± 0.8%. Figure 6b depicts the FFNN emotional classifier CM, which reports the accuracy for each class. 

The McNemar–Bowker test, performed to compare the FFNN classification outcome with the multimodal RF classifier, revealed no statistically significant difference between the two outcomes (B = 3.9, p = n.s.).

#### Features Analysis Results

**Figure 7:**
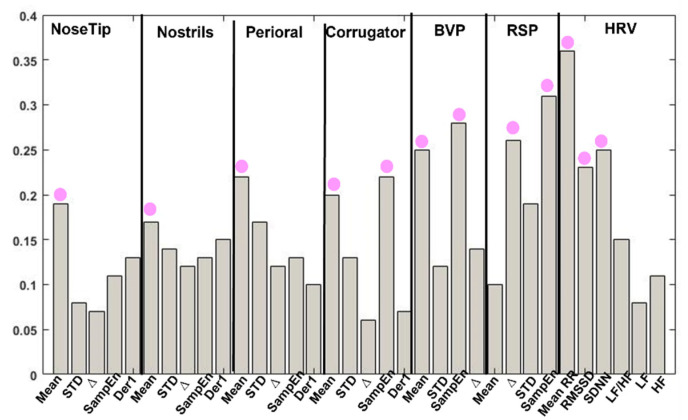
First FeedForward Neural Network layer’s weights. The red dots represent the features associated with the highest weights for each physiological signal.

The FFNN first layer’ weights were retrieved (Figure 7) and evaluated to assess the salient features used by the FFNN to perform its final classification decision. 

**Figure 8:**
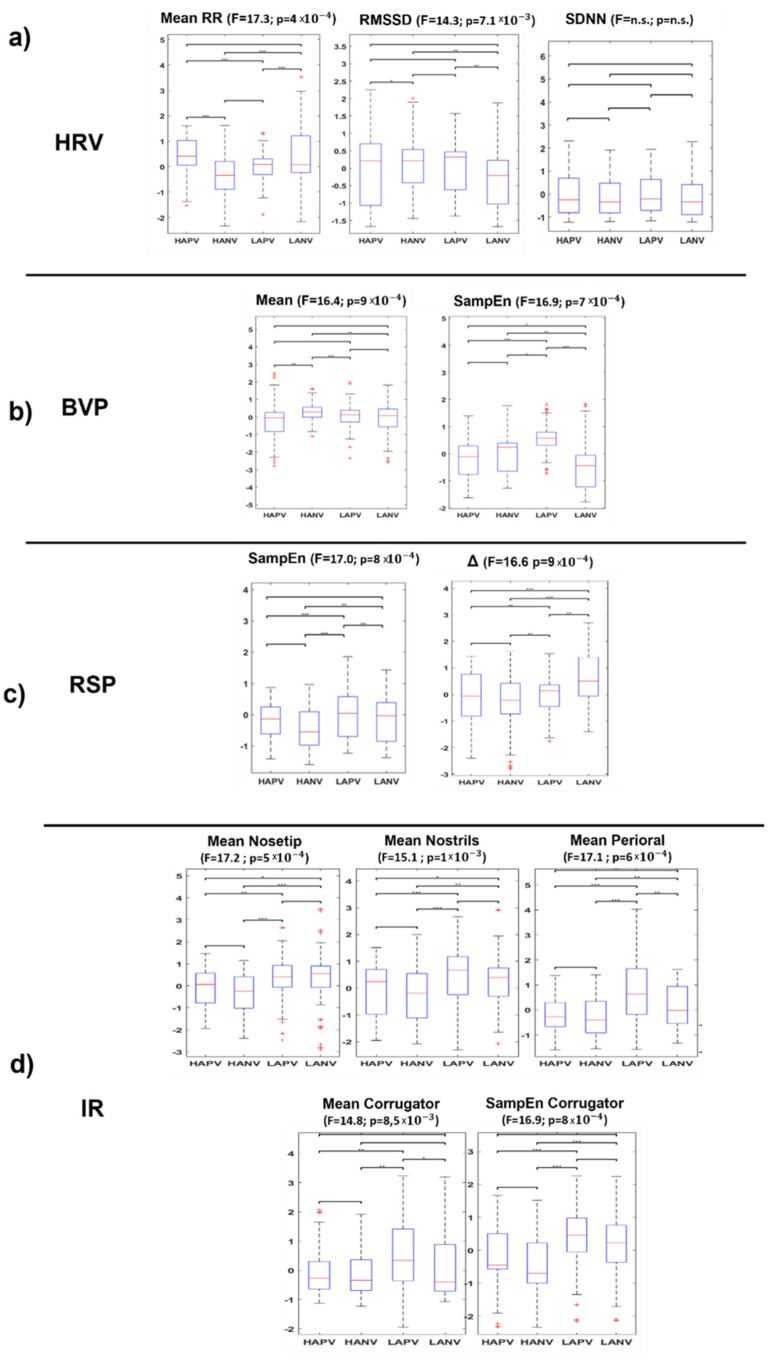
Feature distribution among the four classification classes (i.e., high arousal positive valence, high arousal negative valence, low arousal positive valence, low arousal negative valence): (**a**) Features related to the Heart Rate Variability signal: mean RR interval, the root-mean-square of successive RR interval differences (RMSSD), and the standard deviation of normal-to-normal interval (SDNN), (**b**) Features related to the Blood Volume Pulse signal: mean and Sample entropy. (**c**) Features related to the Respiratory signal: Sample Entropy and signal variation (Δ), and (**d**) Features related to the Infrared signals: mean value of the nose tip, perioral and corrugator area, and sample entropy of the corrugator region. The boxes within each feature plot show the median and interquartile range, with the dashed line indicating the range of values. The title of each feature’s plot reports the repeated measure ANOVA outcome, whilst the results of the multiple comparisons are denoted by asterisks. * *p* < 0.05 ** *p* < 0.01; *** *p* < 0.001 for pairwise comparisons between the classification classes.

These features were then analyzed to investigate the presence of a statistical difference between their trends across the four emotional conditions (i.e., HAPV, HANV, LAPV, LANV). The ANOVA RM results and the multiple comparison outcomes are shown in Figure 8. The resulting p-values were further corrected using Bonferroni correction.

## 4. Discussion

The purpose of this study was to develop an automated emotion recognition model which relied entirely on physiological signals that may be recorded using easy-to-wear or even contactless devices. Indeed, in addition to physiological signals that have been extensively researched for emotion recognition purposes, such as cardiac and respiratory signals, contactless technology (i.e., thermal IR imaging) has been used to classify human emotions.

To evaluate the advantage of the multimodal procedure, three different RF classifiers were implemented and compared. Specifically, a comparison was performed among the classifiers trained on only cardiac features, the classifier based on both cardiac and respiratory features, and the classifier trained on cardiac, respiratory, and thermal features. The overall accuracy of these classifiers measured 59.5%, 63%, and 66%, respectively. Although the accuracy differences were revealed to be not statistically significant, the multimodal strategy outperformed the monomodal approach, depicting the added value of the multimodal procedure. Moreover, it confirmed the thermal IR imaging technique as an important source of information in the emotion recognition tasks. 

Given the higher performance of the multimodal approaches, it was possible to employ innovative ML algorithms designed to manage a vast amount of data, such as FFNN. The FFNN model implemented provided higher accuracy compared to the canonical ML approach. Specifically, the overall accuracy reached by the tuned FFNN model measured 70%. Notably, the classifier’s accuracy was related to the four CMA’s quadrants, which are determined by the respective levels on both valence and arousal dimensions. The sound performance of the model was further confirmed by the CM, which also provided the accuracy reached in each classification class, i.e., 64% in the LANV class, 69% in the HANV class, 71% in the HAPV class, and 74% in the LAPV class.

Focusing on the developed FFNN model, the salient features for the purpose of the classification of each recorded physiological signal were investigated. This was possible by retrieving the weights of the first FFNN layer associated with each input feature. The features associated with the higher weights related to the HRV signal were the mean RR, RMSSD, and SDNN. This result is, indeed, in line with Shi et al. research which showed significant emotional-related changes for time-domain HRV indices (i.e., mean RR, RMSSD, and SDNN) but not significant variation for frequency-domain indices (i.e., LF, HF, LF/HF) [69]. However, one-way ANOVA RM revealed significant differences in the distribution of the features among the four-classification classes only for the mean RR and RMSSD features. Concerning the BVP signal, the salient features were the mean and the SampEn, which is in line with [70]. For the RSP signal, the features resulted to be the SampEn and Δ. In fact, according to Pan et al., nonlinear features based on entropy (e.g., SampEn) of physiological signals such as RSP can represent emotion characteristic in depth [71]. Regarding the IR signals, for the nose tip, nostrils, and perioral ROI, the important feature was the mean value, for the corrugator ROI the salient features were the mean and the SampEn. The one-way ANOVA RM confirmed a statistically significant difference in the distribution of such features among the four-classification class. Moreover, focusing on the IR signals, the analysis showed that such signals seem to be more sensitive to the arousal dimension [72]. Indeed, the multiple comparisons revealed that the statistical difference was mostly due to differences in arousal.

Importantly, this study suggested that DL methodologies were appropriate for modeling affective states from easily accessible physiological signals. This novel approach might be employed in situations where real-time feedback is needed. Moreover, the market entry of smaller and low-cost thermal cameras is paving the way for thermal IR imaging applications outside laboratory environments, especially for cutting-edge applications in the affective computing field. Furthermore, smart thermal devices have proved effective in important real-life scenarios, such as driver drowsiness state evaluation [73,74], human stress recognition [75], and smartphone-based clinical imaging systems [76,77]. In this regard, this research reports a significant novelty. Indeed, although the suitability of thermal imaging to differentiate between emotional states has already been demonstrated [78], this study provides an already validated and ready to use automated emotion recognition model. The model developed by combining thermal imaging with other physiological signals and FFNN algorithms outperforms existing models, such as that reported in Sarath et al. [79]. 

### Study’s Limitations

Finally, despite the highly promising results, it is worth mentioning that this study presents some limitations, such as, for instance, the low sample size. Indeed, the DL classification procedure adopted, relied on a supervised learning approach. Since this approach is inherently data-driven, the sample size bears a significant impact on its performance. However, although the sample size of the study could be considered rather small, the classification was carried out implementing a leave-one-subject-out cross-validation procedure, which essentially evaluated out-of-sample performance. Consequently, the results obtained can be indeed considered generalizable. By reducing the possibility of in-sample overfitting, increasing the sample numerosity may boost the classifier’s performance even further. In addition, the application of DL to model affect in large physiological datasets would potentially exhibit larger improvements with respect to canonical ML or statistical features and provide new insights on the relationship between physiology and affect. Moreover, it would be worth increasing the number of FFNN regressors; however, this solution could introduce an overfitting effect in the learning procedure from the data, which could be avoided by employing large sample sizes.

## 5. Conclusions

This research demonstrates automated emotion recognition from unobtrusive physiological signals recording is possible, thus, paving the way for real-time affective computing in potentially real-life applications. Compared to trait-based information, physiological signals bear the benefit of not being within the person’s control; hence, they do not involve bias in the detection of the individual’s emotional state. Of note, the classification was based on the four CMA quadrants, which considered both arousal and valence dimensions. Different classification procedures were compared, including monomodal and multimodal approaches, as well as ML and DL algorithms. The higher classification accuracy was reached by the DL approach using a multimodal strategy. Specifically, the overall accuracy among the four classification classes measured 70%. Importantly, combined with physiological signals widely researched for emotion detection applications, such as cardiac and respiratory signals, a contactless technology was employed to develop a high accurate emotion classification DL model. The thermal IR imaging technique, indeed, revealed an important source of information in the emotion classification task. Moreover, due to its contactless and agile nature, the IR imaging technique bears the potential to pave the way for innovative applications in the field of affective computing. Finally, although this result suggested that DL approaches are appropriate for automated emotion detection, further studies should be conducted to increase the sample size of the population. 

## Figures and Tables

**Table 1:** Time domain HRV indices.

Variables	Formula
Mean RR	$\mathrm{Mean}=\frac{1}{N}\overset{N}{\underset{i=1}{\sum }}RR_{i}$
RMSSD	$\mathrm{RMSSD}=\sqrt{\frac{\sum_{i=1}^{N-1}{\left(RR_{i+1}-RR_{i}\right)}^{2}}{N-1}}$
SDNN	$\mathrm{SDNN}=\sqrt{\frac{\sum_{i=1}^{N}{\left(RR_{i}-meanRR\right)}^{2}}{N-1}}$

*N* = number of RR interval.

**Table 2:** Description of the features extracted from HRV, BVP, RSP, and IR signals.

Signals	Features *	Description
HRV	LF	Power in low frequency range (0.04–0.15 Hz)
	HF	Power in high frequency range (0.15–0.4 Hz)
	LF/HF ratio	LF/HF
	Mean RR	mean RR interval
	RMSSD	root-mean-square of successive RR interval differences
	SDNN	Standard deviation of normal-to-normal interval
BVP, RSP, IR (× 4 ROIs)	STD	Signal’s standard deviation
	Mean	Signal’s average value
	Δ	Difference between the signal’s average value during task and rest
	SampEn	Sample entropy, which measures the regularity and complexity of a time series
IR ( 4 ROIs)	Der1	Signal’s first-time derivative, which describes the signal tangent line slope

* The total number of features analyzed is 34.

## Data Availability

The data presented in this study are available on request from the corresponding author. The data are not publicly available due to privacy issues.
